# Panic buying in response to tariffs: a psychodynamic and psychoanalytic hypothesis for economic uncertainty

**DOI:** 10.3389/fpubh.2026.1893778

**Published:** 2026-09-18

**Authors:** Marty Cooper

**Affiliations:** 1 State University of New York Old Westbury, Old Westbury, NY, United States; 2 New York University Postdoctoral Program in Psychotherapy and Psychoanalysis, New York, NY, United States

**Keywords:** economic uncertainty, panic buying, psychodynamic, rational stockpiling, tariffs

## Abstract

Panic buying, the sudden, affectively driven surge in purchasing essential goods in excess of rational need, has been widely documented during health crises but remains underexplored as a response to economic policies such as tariffs. Critically, panic buying must be distinguished from rational stockpiling: while anticipatory purchasing in response to tariff-induced price increases may reflect sound intertemporal decision-making, panic buying is characterized by purchases exceeding individually optimal levels and driven by negative affect, perceived scarcity, and social contagion rather than objective risk assessment alone. This article extends the integrated psychodynamic model of panic buying developed by Cooper and Gordon (1) to address tariff-induced consumer behavior, clarifying its psychological underpinnings and broader significance. The article examines how well-established frameworks from behavioral economics, including Prospect Theory, scarcity heuristics, and affect-based models, account for much of the surface phenomenology of tariff-induced panic buying, while arguing that psychodynamic mechanisms explain a clinically significant residual: namely, why a subset of individuals exhibits panic buying that is disproportionate to objective threat, resistant to rational persuasion, and recurrent across successive economic crises. The article introduces three core psychodynamic mechanisms operating across a five-phase model: (1) developmental vulnerability rooted in economic trauma; (2) threat activation involving projection and splitting; and (3) behavioral enactment via displacement and transitional object dynamics. Each mechanism is distinguished conceptually, linked to specific phases of the model, and operationalized with proposed measurement strategies. By providing a clinically actionable framework that complements existing behavioral and economic accounts, the analysis contributes to both therapeutic practice and public policy during periods of economic uncertainty.

## Introduction

Panic buying, defined as a sudden surge in the purchase of essential goods in response to perceived threats or adverse events, characterized by purchases exceeding individually optimal levels and driven by heightened negative affect, has emerged as a significant behavioral phenomenon during crises such as pandemics, natural disasters, and major policy changes (1, 2). While panic buying has been extensively documented during the COVID-19 pandemic, its occurrence in response to economic policies, specifically tariffs, remains underexplored in the psychological literature. Tariffs, which are government-imposed duties on imported goods, can lead to price increases and supply chain disruptions, fostering uncertainty and fear among consumers that closely parallel the psychological conditions precipitating panic buying during health emergencies.

Existing research has established that panic buying is driven by negative emotions such as anxiety, fear, and institutional mistrust, particularly during periods of uncertainty or perceived scarcity (3, 4). These emotions are further amplified by media coverage and social contagion, leading to atypical consumer behaviors that disrupt supply and demand. The psychodynamic framework, as articulated by Cooper and Gordon (1), conceptualizes panic buying as a defense mechanism rooted in developmental history, unresolved trauma, and the need to regain control during destabilizing events.

Despite growing interest in the psychological underpinnings of panic buying, there is a notable gap in the literature regarding its occurrence in response to tariffs and other economic policy shifts. Most studies have focused on health crises, with limited attention to the unique triggers and psychodynamic processes associated with economic stressors. This gap is significant: tariffs evoke similar patterns of uncertainty, mistrust, and perceived scarcity, potentially activating deep-seated anxieties and maladaptive coping strategies. Moreover, tariff-induced panic buying unfolds over a longer temporal arc than acute health crises, posing distinct challenges for both therapeutic intervention and public policy response.

The present article addresses this gap by extending the psychodynamic conceptualization of panic buying to include responses to tariffs. Grounded in Cooper and Gordon’s (1) integrated six-phase treatment model, the article argues that while behavioral economic frameworks explain much of the surface phenomenology of panic buying, psychodynamic mechanisms explain a clinically significant residual, particularly the subset of cases characterized by disproportionate severity, resistance to rational reassurance, and recurrence. Attention is also given to the role of socioeconomic status and cultural context, which have been underrepresented in existing frameworks. In doing so, this work aims to inform therapeutic interventions and contribute to the broader discourse on consumer behavior during economic crises.

## Distinguishing panic buying from rational stockpiling

A critical preliminary step, raised by recent consumer behavior research, is distinguishing panic buying from rational or strategic stockpiling. When consumers reasonably anticipate that tariffs will increase prices for durable goods or non-perishables, purchasing larger quantities in advance constitutes an economically rational intertemporal consumption decision (5, 6). Such behavior reflects standard forward-looking optimization: the consumer correctly perceives a future price increase and acts to minimize total expenditure. This is stockpiling, not panic buying.

Panic buying, by contrast, is defined by three features that distinguish it from rational stockpiling: (1) purchases exceed the individually optimal quantity required to address the expected shortage or price increase; (2) the behavior is driven primarily by heightened negative affect, anxiety, fear, and perceived loss of control, rather than accurate risk assessment; and (3) the behavior is responsive to social contagion and media narratives independent of objective supply data (2, 7). This distinction has direct empirical implications: rational stockpiling should track objectively announced tariff levels and expected price changes, whereas panic buying should correlate with measures of consumer anxiety, media exposure, and institutional distrust over and above objective economic indicators.

Importantly, the same purchasing behavior, buying larger-than-usual quantities of a product, may reflect rational stockpiling in one consumer and panic buying in another, depending on the underlying psychological processes. It is precisely this within-behavior heterogeneity that psychodynamic frameworks are particularly equipped to address: by examining the developmental histories, characteristic defense mechanisms, and affective states of individual consumers, clinicians can identify which individuals are engaging in disproportionate, affectively driven purchasing and intervene accordingly. A necessary corollary of this framework is that “disproportionality” as a criterion requires an explicit, independently specified benchmark of individually optimal purchase quantity — for instance, the quantity justified by announced tariff levels, expected price changes, household consumption rates, and storage capacity. Without such a benchmark, the residual that psychodynamic mechanisms are proposed to explain risks being defined negatively, as whatever behavioral economic models fail to predict, rather than measured independently. Future empirical work operationalizing this framework should specify and validate such a benchmark *a priori*, so that the psychodynamic contribution can be assessed against a defined standard rather than a theoretical remainder.

## Theoretical background

### Competing frameworks and the unique contribution of psychodynamic theory

Before introducing the psychodynamic framework, it is necessary to consider what established frameworks from behavioral economics, decision science, and consumer psychology already explain, and where they leave explanatory gaps that a psychodynamic account fills.

Prospect Theory (8) offers a parsimonious account of many panic buying behaviors. Loss aversion predicts that consumers will respond more intensely to the prospect of losing access to goods (through scarcity or price increases) than to equivalent potential gains, and that losses will be experienced as roughly twice as painful as equivalent gains are pleasurable. Applied to tariff contexts, Prospect Theory explains why consumers overreact to tariff announcements relative to the objective probability and magnitude of price increases. However, Prospect Theory does not explain individual differences in the severity of the response: why do some consumers exhibit buying behavior far exceeding what loss aversion alone would predict, while others, facing identical objective conditions, do not panic buy at all?

Scarcity heuristics and availability-based cognition offer complementary accounts of how media framing of shortages creates perceived scarcity that drives purchasing independent of actual supply conditions (9). Affect-based models of decision-making demonstrate that negative emotional states systematically bias risk perception and choice toward protective behaviors (3). Herd behavior and social contagion models explain how observing others stockpile creates normative pressure to do likewise, regardless of individual need (3). Together, these frameworks provide a robust account of the aggregate pattern of tariff-induced panic buying.

What they do not explain is the clinically significant subset of panic buying behavior that is: (a) disproportionate to the objective economic threat as defined by any rational model; (b) resistant to accurate information about supply conditions, which should, on a rational or heuristic model, reduce panic; (c) recurrent across successive economic crises in the same individuals, suggesting a stable psychological vulnerability rather than a situational response; and (d) associated with significant psychological distress beyond the buying behavior itself, such as generalized anxiety, somatic symptoms, or relationship strain. It is precisely this residual, unexplained by behavioral economic frameworks, that psychodynamic theory is uniquely positioned to address, by tracing the behavior to developmental histories and unconscious processes that operate independently of rational appraisal.

### Panic buying in economic contexts: from health crises to trade policy

Existing empirical models of panic buying emerged predominantly from health crises, particularly COVID-19, where supply chain disruptions and existential threats triggered widespread hoarding behaviors (1, 2). Tariff-induced panic buying shares these core psychological drivers while diverging in its economic triggers and temporal dynamics. Tariffs create anticipatory scarcity: consumers fear future price increases or shortages before disruptions materialize (10). Unlike pandemic-driven buying, which centers on immediate survival needs, tariff-related panic buying often targets durable goods and non-perishables, reflecting a response to perceived long-term economic instability. Research on dysfunctional safety behavior during health crises further demonstrates how fear and perceived threat reliably produce hoarding patterns that parallel those expected in tariff contexts (11).

The psychological processes driving tariff-related panic buying include uncertainty amplification through media coverage of trade policy developments (4); projection of systemic mistrust, whereby consumers externalize fears onto governments or foreign entities, viewing tariffs as evidence of institutional incompetence or betrayal (2); and social contagion, in which observing others stockpile creates normative pressure that reinforces buying as a socially sanctioned coping strategy (3). While direct empirical documentation of tariff-induced panic buying as distinct from rational stockpiling remains limited, the psychological conditions that produce panic buying in health-crisis contexts, uncertainty, institutional mistrust, social contagion, and negative affect, are plausibly present when major tariff announcements are made, and the parallel is sufficiently close to warrant hypothesis generation and empirical investigation.

### Psychodynamic foundations: Cooper and Gordon’s integrated model

Cooper and Gordon’s (1) integrated psychodynamic model identifies panic buying as a defense mechanism against feelings of helplessness and existential threat. Their framework emphasizes how developmental histories marked by resource deprivation or economic trauma increase vulnerability to regressive behaviors during destabilizing events. The model comprises six clinical phases: (1) intake, where current symptoms and developmental history are gathered; (2) identification of related themes between history and current presentation; (3) exploration of how the current context activates themes from developmental history; (4) psychoeducation including mindfulness training; (5) skill acquisition and rehearsal; and (6) consolidation and termination.

The five-phase model proposed in the present article (Table 1) is a descriptive-phenomenological framework, mapping the sequence of psychological events from pre-existing vulnerability through behavioral enactment and reinforcement, that operates in parallel with, and is distinct from, Cooper and Gordon’s (1) six-phase clinical treatment model. The five phases describe what happens psychologically during a tariff-induced panic buying episode; Cooper and Gordon’s six phases describe what the clinician does in response. The two frameworks are complementary and sequentially related: clinical phases 1–2 correspond to assessment of the phenomenological phases 1–2; clinical phases 3–5 address the mechanisms active in phenomenological phases 3–4; and clinical phase 6 addresses the prevention of re-entry into the phenomenological phase 5 reinforcement cycle.

**Table 1:** Integrated multiphase model of tariff-induced panic buying: Mechanisms, interventions, and proposed measures.

Phase	Psychodynamic mechanism	Dominant defense(s)	Clinical interventions	Policy interventions	Proposed measures
1. Developmental Vulnerability	Childhood economic trauma encodes persistent threat schemas and scarcity sensitivity	Regression; Repetition Compulsion	Genogram analysis; developmental history intake; psychoeducation linking past and present economic anxiety	Targeted outreach to historically marginalized communities with histories of resource insecurity	ACEs scale adapted for economic trauma; Economic Hardship Questionnaire (EHQ)
2. Trigger	Tariff announcements activate threat appraisal; policy language perceived as confirming institutional unreliability	Projection; Splitting	Reality testing; cognitive restructuring of catastrophic appraisals of policy announcements	Clear, jargon-free governmental communication; proactive supply-chain transparency statements	State anxiety measures (STAI) proximate to tariff announcement dates; institutional trust scales
3. Amplification	Media and social contagion escalate perceived scarcity; normative pressure frames stockpiling as prudent	Displacement; Splitting	Mentalization techniques; media literacy psychoeducation; distinguishing rational from panic-driven appraisal	Behavioral nudge campaigns; purchase-pace transparency; correction of scarcity misinformation	Consumer sentiment indices; social media sentiment analysis; purchasing frequency data
4. Behavioral	Stockpiling enacted as displacement of existential anxiety; hoarded goods function as transitional objects providing felt security	Displacement; Transitional Object Use	Explore symbolic meaning of hoarded goods; MBCT to anchor in present; explore illusion of control	Purchase limits to interrupt cascade; real-time inventory data to reduce perceived scarcity	Purchase quantity relative to baseline; Panic Buying Scale [PBS; (4)]
5. Reinforcement	Actual shortages created by panic buying validate initial fears; feedback loop entrenches the behavior	Repetition Compulsion	Consolidation of adaptive coping; termination planning; relapse prevention for future economic events	Policy monitoring of demand spikes; anti-hoarding communication; supply stabilization measures	Longitudinal purchasing data across tariff cycles; anxiety recurrence measures post-shortage

Clinical interventions refer to individual-or group-level therapeutic approaches. Policy interventions refer to governmental or societal-level responses. Proposed measures are offered as starting points for empirical operationalization; validated adaptations for economic contexts will require further psychometric development. ACEs, Adverse Childhood Experiences scale; EHQ, Economic Hardship Questionnaire; STAI, State–Trait Anxiety Inventory; PBS, Panic Buying Scale (4).

### Limitations of current models and the need for extension

While Cooper and Gordon’s (1) framework provides a robust clinical scaffold, three gaps are particularly salient in economic contexts. First, regarding temporal dynamics: most models focus on acute crises, whereas tariff effects unfold over months or years, complicating both assessment and intervention timing. Second, regarding class and cultural variability: current frameworks underrepresent how socioeconomic status and cultural norms, for instance, collectivist versus individualist orientations, modulate responses to trade policies. Third, regarding policy feedback loops: few studies examine how panic buying itself influences tariff outcomes, such as artificially inflated demand that may justify prolonged trade barriers, deepening economic distrust in a self-reinforcing cycle (12).

### Central hypothesis

The primary hypothesis advanced in this article is that a clinically significant subset of consumer purchasing behavior following tariff announcements constitutes panic buying, as distinguished from rational stockpiling, and that this panic buying is generated by a psychodynamic process in which tariff-related economic threat activates unconscious defenses rooted in developmental histories of resource deprivation, producing affectively driven purchasing behavior that exceeds individually optimal levels and is resistant to rational reassurance.

Three secondary, testable hypotheses follow from this framework. First, the severity of panic buying in response to tariff announcements will be predicted by measures of developmental economic trauma (e.g., childhood exposure to poverty, food insecurity, or economic instability) over and above objective indices of tariff impact, consumer anxiety, and media exposure. Second, specific psychodynamic processes will differentially mediate the relationship between tariff threat and panic buying: projection and splitting will mediate the relationship between institutional mistrust and threat amplification; displacement will mediate the relationship between felt helplessness and purchasing behavior; and transitional object dynamics will moderate the relationship between attachment insecurity and stockpile quantity. Third, panic buying severity will predict recurrence across successive tariff-related events in the same individuals, consistent with repetition compulsion, whereas rational stockpiling will not show this cross-event stability after controlling for objective economic conditions.

These hypotheses are distinguishable from predictions generated by behavioral economic frameworks: Prospect Theory predicts that loss aversion, not developmental history, will be the primary predictor of response magnitude; scarcity heuristics predict that media framing, not attachment insecurity, will drive stockpile quantity. The psychodynamic framework generates unique predictions that can be falsified independently of these alternatives.

### Three core psychodynamic mechanisms in tariff-induced panic buying

The present framework consolidates the psychoanalytic literature into three core mechanisms, each conceptually distinct, operating at different phases of the model, and generating distinct empirical predictions and clinical implications. This consolidation responds to the concern that a proliferation of constructs without clear differentiation weakens explanatory parsimony. While the broader psychoanalytic literature offers many relevant concepts, the three mechanisms below are selected because they address different phases of the panic buying sequence: developmental vulnerability, threat activation, and behavioral enactment.

### Mechanism 1: developmental vulnerability — regression and repetition compulsion

The first mechanism operates at the developmental phase, prior to any specific tariff event. Individuals who experienced significant economic hardship during formative years, poverty, food insecurity, parental financial instability, may develop persistent threat schemas in which resource scarcity is encoded as a proximate and recurring danger. When confronted with tariff-related economic news, these individuals are prone to regression: a return to earlier modes of functioning in which primitive accumulation behaviors (hoarding) were adaptive responses to genuinely scarce resources (13). Critically, this regression is typically not experienced consciously; the individual perceives their stockpiling as rational precaution rather than as a re-enactment of earlier coping strategies.

Repetition compulsion, the unconscious tendency to re-enact unresolved past experiences in an attempt to master them, provides a complementary account of why this vulnerability manifests as a stable, cross-situational pattern (13). Consumers with developmental economic trauma do not engage in disproportionate panic buying because each new tariff episode is uniquely threatening; they do so because the pattern of response is encoded from earlier experiences and is repeatedly activated by cues of economic threat. This explains the recurrence of panic buying in the same individuals across multiple crisis episodes, a pattern that loss aversion and scarcity heuristics do not predict once objective conditions are controlled. Empirically, this mechanism is operationalizable through the Adverse Childhood Experiences (ACEs) scale adapted for economic trauma, or the Economic Hardship Questionnaire, administered in conjunction with longitudinal purchasing data.

### Mechanism 2: threat activation — projection and splitting

The second mechanism operates at the trigger and amplification phases, mediating between the receipt of tariff-related information and the intensity of the threat response. Projection involves the externalization of internal states, particularly fears of inadequacy, helplessness, and vulnerability, onto external objects or actors. In tariff contexts, consumers project their own anxieties onto governmental institutions or foreign trading partners, experiencing tariff policies as hostile or incompetent acts by untrustworthy others rather than as routine policy instruments with complex intended and unintended consequences (2). This projection amplifies the perceived threat and generates what is clinically recognizable as paranoid anxiety about economic actors, anxiety that exceeds what objective analysis of trade policy would warrant.

Splitting, the tendency to perceive institutions and situations in polarized, all-or-nothing terms, operates in conjunction with projection to create a cognitive-affective environment in which panic is both more likely and more intense. In tariff contexts, splitting manifests as rigid dichotomization: domestic goods are idealized, foreign goods or trading partners are demonized, one’s government is experienced as either wholly reliable or wholly malevolent. This black-and-white cognition forecloses the nuanced, probability-weighted appraisal that characterizes rational stockpiling. Crucially, projection and splitting are conceptually distinct from the affect-based decision models of behavioral economics: whereas affect-based models treat negative emotion as an input that distorts otherwise rational cognition, the psychodynamic account treats projection and splitting as motivated, structured distortions organized around specific relational and developmental anxieties. Empirically, this mechanism is operationalizable through institutional trust scales administered proximate to tariff announcements, combined with measures of splitting (e.g., the Splitting Index or object relations measures) and anxiety (STAI).

### Mechanism 3: Behavioral enactment — displacement and transitional object dynamics

The third mechanism operates at the behavioral phase, explaining how threat activation is translated into specific purchasing behavior. Displacement involves redirecting anxiety from its true source, diffuse economic insecurity and felt helplessness in the face of macroeconomic forces beyond individual control, toward a manageable behavioral outlet. Stockpiling provides the illusion of agency: the individual cannot control tariff policy but can control the contents of their pantry. This mechanism is conceptually distinct from the herd behavior and social contagion accounts offered by behavioral economics, which explain why individuals conform to others’ purchasing behavior; displacement explains why individuals who are not influenced by social contagion, and who may even recognize their purchasing as excessive, continue nonetheless, because the behavior functions to manage an internal state rather than respond to an external norm.

Transitional object dynamics (14) provide the most clinically generative account of the specific form that displaced anxiety takes in panic buying. Winnicott’s concept of transitional objects, physical embodiments of security that provide comfort during separation or threat, illuminates why stockpiled goods carry affective significance beyond their practical value. The hoarded cans of food or cases of household goods are not merely rational preparations; for the developmental-vulnerability subset, they are symbolic containers for anxieties that cannot be more directly addressed. This framing generates a unique clinical prediction: the symbolic content of hoarded goods (their specific categories and quantities) will be more strongly predicted by attachment insecurity and felt threat to caregiving security than by objective measures of supply disruption or price change. Empirically, this mechanism is operationalizable through the Panic Buying Scale (PBS; 4), supplemented by measures of attachment style and items assessing the felt security provided by stockpiled goods.

### From clinical observation to population-level phenomena: scope and limits of the framework

A central question raised by any extension of clinical psychoanalytic concepts to consumer behavior is whether mechanisms originally derived from individual therapeutic observation can legitimately be applied to population-level patterns. This concern is well-founded and warrants explicit address.

The present framework does not claim that all panic buying, or even most panic buying, is explained by psychodynamic mechanisms. As the section on competing frameworks makes clear, prospect theory, scarcity heuristics, affect-based models, and social contagion explain a substantial portion of aggregate panic buying behavior. The psychodynamic framework advances a more limited claim: that within any population of consumers responding to a tariff announcement, there is a clinically significant subset for whom the severity, persistence, and resistance to rational reassurance of their panic buying behavior reflects the operation of developmentally rooted unconscious processes. This subset will be identifiable by their scores on the proposed measures (developmental economic trauma, attachment insecurity, institutional distrust, splitting) and will exhibit the specific patterns of recurrence and disproportionality that the three mechanisms predict.

This claim is empirically tractable at the population level. Structural equation modeling or multi-level modeling applied to consumer panel data, combining purchasing behavior with the proposed psychological measures, could test whether developmental economic trauma and attachment insecurity predict panic buying severity over and above Prospect theory variables (loss aversion, reference-point shifts) and social contagion indicators. Survey-based experimental designs could expose participants to varying tariff announcement framings and examine whether the psychodynamic predictors moderate response severity differentially across framing conditions.

The framework also has implications beyond the individual clinical encounter. If a predictable subset of the consumer population is prone to disproportionate panic buying due to psychodynamic vulnerability, then population-level interventions, such as governmental communication strategies, can be calibrated to address not only rational stockpiling but also the affective and relational dimensions that drive the panic-buying subset. Governments communicating about tariff impacts, for instance, should attend not only to the accuracy of their supply projections but also to how the framing of institutional reliability (countering projection) and supply adequacy (reducing splitting) is received by consumers with high institutional distrust.

### An integrated multiphase model for tariff-induced panic buying

Table 1 presents the integrated five-phase model, incorporating the three core psychodynamic mechanisms described above. Each phase is linked to its dominant psychological mechanism, the specific defense or process active at that phase, separate clinical and policy interventions, and proposed empirical measures. As noted in the Theoretical Background, this five-phase descriptive model maps the phenomenological sequence of a panic buying episode and is complementary to, not a replacement for, Cooper and Gordon’s (1) six-phase clinical treatment model.

The model highlights the interplay between macro-level policy and micro-level psychodynamics. Critically, the Reinforcement phase incorporates the policy feedback loop that is largely absent from clinical models: panic buying artificially inflates demand and can create real shortages where none initially existed, thereby validating consumers’ initial fears and perpetuating the cycle (12). This feedback mechanism implies that clinical intervention at the Behavioral phase, before reinforcement is established, is more effective than intervention after the cycle has consolidated, and that policy-level interruption of demand spikes is both an economic and a mental health intervention.

### Socioeconomic and cultural dimensions

Economic anxiety and risk aversion are heightened during periods of policy uncertainty, influencing both consumer behavior and broader political attitudes (15). Crucially, socioeconomic status may modulate both the objective exposure to tariff impacts and the psychological vulnerability described in Mechanism 1. Individuals and families with lower incomes face greater material exposure to tariff-induced price increases and less financial resilience to absorb them, amplifying the psychological urgency of stockpiling. At the same time, the practical capacity to stockpile is itself a function of economic privilege: buying in bulk, having storage space, and having access to transportation and cash are resources differentially available to more affluent consumers. Panic buying can therefore be paradoxically more psychologically pressing yet more materially constrained for those with fewer resources, a dynamic with implications for how population-level interventions are designed and targeted.

Cultural context shapes the phenomenology of tariff-induced panic buying in ways that modulate the three core mechanisms. In collectivist cultural frameworks, the social contagion dimension of the Amplification phase is likely intensified: the psychological imperative to protect one’s household by conforming to community behavior carries greater moral weight, and failure to stockpile may generate social shame as well as personal anxiety. In individualist frameworks, displacement dynamics may be intensified, as the inability to single-handedly secure one’s family against macroeconomic forces is experienced as a personal failure. These cultural differences in phenomenology suggest that the relative weight of the three mechanisms may vary cross-culturally, a prediction that structured cultural comparison studies could examine.

Political distrust and the anticipation of governmental inadequacy further fuel the projection and splitting mechanisms, as consumers externalize economic insecurity onto institutional actors perceived as unreliable (12). For populations with histories of systemic oppression or disproportionate exposure to negative governmental action, for whom institutional distrust is historically grounded rather than merely dispositional, tariff policies may carry layered meanings that intensify threat activation independent of developmental economic trauma. Clinicians should remain attentive to this distinction: not all institutional distrust reflects psychopathological projection; some reflects accurate historical appraisal, and interventions should be calibrated accordingly.

### Clinical applications

The integrated model suggests phase-specific clinical approaches that build on Cooper and Gordon’s (1) six-phase treatment framework. During intake, genogram analysis can identify intergenerational patterns of economic trauma and resource anxiety, operationalizing the developmental vulnerability assessed in Phase 1. Psychoeducation that normalizes the psychological response to economic uncertainty, framing it as an understandable response to developmental history rather than a pathological defect, can reduce shame and increase engagement.

At the Trigger and Amplification phases, the identification of projection and splitting provides specific therapeutic targets. Reality testing, examining the evidence for and against catastrophic appraisals of tariff impacts, addresses projection. Mentalization techniques that support clients in holding complexity and ambiguity, in their perceptions of governmental actors, foreign trading partners, and economic uncertainty, address splitting. Both approaches are consistent with Cooper and Gordon’s (1) emphasis on working through interpersonal dynamics and correcting cognitive distortions.

At the Behavioral phase, the transitional object framing provides a clinically generative entry point. Exploring what stockpiled goods mean to the client, what they feel like they provide, what the client imagines would happen without them, opens inquiry into the underlying attachment history and developmental deprivation that drives the behavior. Mindfulness-based cognitive therapy (MBCT), as proposed by Cooper and Gordon (1), is particularly suited to displacement: anchoring clients in present-moment experience interrupts the future-oriented, anticipatory anxiety that drives anticipatory stockpiling and helps clients distinguish current reality from past deprivation.

Identifying regression and repetition compulsion across the clinical history, by examining whether the client has engaged in similar disproportionate purchasing behaviors during previous economic or personal crises, provides evidence for the developmental mechanism and enables psychoeducation about the cross-situational pattern. This historical perspective can motivate engagement with the developmental work that distinguishes psychodynamic treatment from purely symptom-focused approaches.

## Discussion

This article has proposed and elaborated a psychodynamic account of panic buying in response to tariffs that is explicitly positioned as complementary to, not a replacement for, well-established behavioral economic frameworks. The central argument is not that prospect theory, scarcity heuristics, and social contagion are insufficient to explain panic buying in general, but that they leave a clinically significant and empirically identifiable residual unexplained, a subset of consumers whose panic buying is disproportionate, recurrent, and resistant to rational reassurance, and that this residual is explicable through three core psychodynamic mechanisms operating across five sequential phases.

The consolidation of the psychoanalytic literature into three mechanisms, developmental vulnerability (regression and repetition compulsion), threat activation (projection and splitting), and behavioral enactment (displacement and transitional object dynamics), addresses the concern that the psychodynamic framework proliferates constructs without sufficient differentiation. Each mechanism operates at a distinct phase, generates distinct predictions, implies distinct clinical interventions, and is operationalizable with distinct measurement strategies. Together they form a coherent mechanistic account rather than a post-hoc interpretive collection.

The temporal dimension of tariff-induced panic buying distinguishes it meaningfully from acute crisis buying and has important clinical implications. Because tariff effects unfold gradually and are announced in advance, there is a pre-event window, between announcement and implementation, during which the Trigger and Amplification phases are active but the Behavioral phase has not yet fully consolidated. Clinical and policy intervention during this window, targeting projection, splitting, and the conditions for transitional object formation, may be more effective than post-hoc intervention after the reinforcement cycle has been established.

It should be acknowledged that this article presents a hypothesis-generating framework rather than an empirical validation. The three mechanisms and the secondary hypotheses derived from them have not yet been empirically tested in tariff contexts. The proposed measurement strategies are offered as starting points; psychometric development specifically for economic crisis contexts is needed. Future research should examine consumer panel data spanning tariff announcement periods, combining purchasing behavior with the proposed psychological measures in structural equation or multi-level designs. Longitudinal studies tracking the same individuals across successive tariff-related events would provide the strongest test of the recurrence predictions derived from the repetition compulsion hypothesis.

### Limitations

Several limitations of this framework should be acknowledged. First, the psychodynamic mechanisms proposed have not been empirically tested in the context of tariff-induced panic buying; the model is hypothesis-generating and should not be understood as empirically validated. Second, the proposed measurement strategies require psychometric development for economic crisis contexts, and the available instruments (ACEs, STAI, PBS) were not designed specifically for this application. Third, the framework is most applicable to cases in which panic buying is clinically significant in severity and distress; many instances of above-baseline purchasing following tariff announcements will reflect rational stockpiling and require neither clinical attention nor psychodynamic interpretation. Fourth, the psychodynamic approach requires more time and therapeutic depth than brief interventions permit, which may limit its applicability in resource-constrained settings. Fifth, while this article argues for the importance of cultural and socioeconomic context in modulating the three mechanisms, it does not provide culturally specific clinical adaptations supported by outcome data, which remains an important direction for future development. Sixth, a methodological tension exists in the operationalization of putatively unconscious processes through self-report instruments: by definition, mechanisms such as regression, projection, and splitting operate below the threshold of conscious awareness, and respondents cannot be expected to report directly on their activation. The proposed measures — attachment style inventories, institutional trust scales, splitting indices — capture constructs associated with these mechanisms rather than the mechanisms themselves, and their validity as proxies for unconscious processes requires explicit psychometric justification in future empirical work. Behavioral and physiological measures, as well as clinician-rated assessments in therapeutic contexts, may provide more direct access to these processes and should be incorporated into research designs alongside self-report.

## Conclusion

The psychological dimensions of panic buying in response to tariffs represent an underexplored but practically significant area for clinical and scholarly attention. This article has extended Cooper and Gordon’s (1) integrated psychodynamic model by consolidating the relevant psychoanalytic literature into three core mechanisms, developmental vulnerability, threat activation, and behavioral enactment, that operate sequentially across five phenomenological phases and generate distinct, falsifiable predictions distinguishable from those of established behavioral economic frameworks.

The framework advances a bounded claim: not that psychodynamic mechanisms account for all panic buying, but that they explain a clinically significant subset characterized by disproportionate severity, resistance to rational reassurance, and cross-event recurrence. Understanding this subset through a psychodynamic lens enables clinicians to address the developmental roots of maladaptive consumer behavior rather than its surface manifestations alone, and enables policymakers to design communications that address not only rational stockpiling but also the affective and relational dimensions that drive panic in economically and developmentally vulnerable consumers. Together, clinical practice and public policy informed by this framework can better prepare individuals and communities for the disruptions that future economic policy changes will bring.

## Data Availability

The original contributions presented in the study are included in the article/supplementary material, further inquiries can be directed to the corresponding author.
